# PSMA expression assessed by [^18^F]PSMA-1007 PET/CT imaging in metastatic hormone-sensitive prostate cancer patients treated with apalutamide

**DOI:** 10.1186/s40644-025-00965-y

**Published:** 2025-12-06

**Authors:** Elena Katharina Berg, Sophie Carina Kunte, Josef Zahner, Adrien Holzgreve, Can Daniel Aydogdu, Hans Peter Schmid, Lennert Eismann, Severin Rodler, Marcus Unterrainer, Rudolf Alexander Werner, Christian Georg Stief, Lena Maria Unterrainer, Jozefina Casuscelli

**Affiliations:** 1 Department of Urology, LMU University Hospital, LMU Munich, Marchioninistr. 15, 81377 Munich, Germany; 2 Department of Nuclear Medicine, LMU University Hospital, LMU Munich, Munich, Germany; 3 Bavarian Cancer Research Center (BZKF), Partner Site Munich, Munich, Germany; 4 Ahmanson Translational Theranostics Division, David Geffen School of Medicine at UCLA, Los Angeles, CA USA; 5 Department of Urology, University Hospital Schleswig-Holstein, Kiel, Germany; 6 Die Radiologie, Munich, Germany; 7 Division of Nuclear Medicine and Molecular Imaging, The Russell H Morgan Department of Radiology and Radiological Science, Johns Hopkins School of Medicine, Baltimore, MD USA

**Keywords:** PSMA, Imaging, Apalutamide, Prostate cancer, ARPI

## Abstract

**Background:**

PSMA PET/CT (Prostate-specific membrane antigen positron emission tomography / computed tomography) has revolutionized prostate cancer imaging. While PSMA expression is influenced by androgen receptor pathway inhibitors (ARPI), few studies have examined the specific effects of apalutamide, a widely used ARPI for hormone-sensitive metastatic prostate cancer. This study evaluated the impact of apalutamide on PSMA radioligand uptake.

**Methods:**

We evaluated 17 patients with metastatic hormone-sensitive prostate cancer (mHSPC) who underwent [^18^F]PSMA-1007 PET/CT imaging prior to (PET1) (time from PET1 to initiation of apalutamide: 0.40 months; range 0.03–5.90 months) and during apalutamide treatment (PET2) (time from apalutamide initiation to PET2: 5.30 months; range 1.70–20.30 months). Biodistribution and tumor uptake were calculated using whole body total tumor volume (TTV), TTV-SUV_max_ and TTV-SUV_mean_ and changes between imaging time points were analysed.

**Results:**

All patients demonstrated significant clinical and biochemical PSA responses after treatment initiation (median PSA (ng/mL) 46.40 vs. 0.03, *p* = 0.001). On PSMA PET/CT during apalutamide therapy, patients showed an overall decrease in TTV-SUV_max_, TTV-SUV_mean_ and TTV. Median decreases in TTV-SUV_max_ and TTV-SUV_mean_ were − 54% and − 28%, respectively (*p* = 0.006, *p* = 0.020). TTV exhibited a significant median decrease by -53% (*p* = 0.006). In 4/17 (23.5%) patients imaging and biochemical response revealed discordant results.

**Conclusions:**

This pilot study indicates that, in mHSPC patients, PSMA upregulation is unlikely with intermediate to long-term apalutamide therapy. However, an increase of imaging parameters with synchronous decrease of PSA levels should raise suspicion of early imaging progression, resulting in close monitoring. Further research is warranted to explore early treatment response in the hormone-sensitive stadium.

## Background

Combination therapies have become the standard primary approach for treating metastatic hormone-sensitive prostate cancer (mHSPC). The second-generation androgen receptor pathway inhibitor (ARPI) apalutamide is a widely utilized therapeutic agent in this context, reducing tumor volume by suppressing tumor cell proliferation and enhancing apoptosis [1]. The phase III TITAN study demonstrated significantly prolonged overall survival and progression-free survival for the combination of apalutamide and androgen deprivation therapy (ADT) compared to placebo and ADT [2], establishing it as a therapeutic option suitable for a broad range of patients. Prostate-specific membrane antigen (PSMA) is a transmembrane glycoprotein overexpressed in prostate cancer cells [3]. Positron emission tomography (PET) targeting PSMA has revolutionized prostate cancer imaging for primary staging and follow-up, offering higher sensitivity and specificity in detecting local and metastatic lesions compared to conventional imaging [3, 4]. Response assessment can be conducted using RECIP, integrating the occurrence of new lesions and changes in PSMA-positive total tumor volume (TTV) [4, 5]. Consequently, most major guidelines have incorporated PSMA PET/CT as a staging modality in high-risk localised, locally advanced and metastatic prostate cancer, whenever available. PSMA radiopharmaceutical therapy (RPT) is a therapeutic option in men with metastatic castration-resistant prostate cancer (mCRPC) [6]. However, PSMA-RPT is not indicated for patients with low PSMA expression, as intense PSMA expression is deemed critical for a potential therapeutic response. Expanding investigations of RPT in earlier settings have raised the question of whether mechanisms can be employed to enhance PSMA expression, thereby creating optimal conditions for RPT. In vitro and in vivo studies have demonstrated that hormonal status can influence PSMA expression [7, 8]. Preliminary evidence suggests that ARPI could induce PSMA upregulation. Preclinical molecular studies were able to detect PSMA upregulation effects following androgen blockade by enzalutamide in prostate cancer cell lines [9]. To date, a limited number of clinical studies has examined the impact of AR-targeting treatment on PSMA expression, with most yielding heterogeneous results [10–13]. It is important to note, that the majority of these studies were conducted in the castration-resistant setting, where tumour biology and treatment effects on PSMA regulation may differ substantially from those observed in hormone-sensitive disease. In contrast, data on PSMA expression dynamics under ARPI treatment in mHSPC remain scarce. This distinction is crucial, as prior findings of PSMA upregulation following ARPI or ADT initiation may not directly translate to the hormone-sensitive setting. Additionally, a time aspect with PSMA increase under short-term ADT and decrease after long-term ADT is mentioned in a subset of trials [13]. A potential upregulation effect has been proposed as a mechanism to ‘prime’ the diagnostic accuracy of PSMA PET and improve the therapeutic efficacy of PSMA-RPT [14].

To our knowledge, effects of apalutamide on PSMA expression in PSMA PET have not yet been analysed. As mentioned above, the majority of previous studies on ARPI and PSMA expression were further focusing solely on mCRPC patients. The aim of this pilot study was to specifically evaluate changes on serial whole-body (WB) PSMA PET parameters among patients with mHSPC in a clinical setting prior to and after initiation of apalutamide.

## Materials and methods

### Study design and patient selection

This retrospective analysis was conducted at a single center, involving patients diagnosed with mHSPC who received apalutamide plus ADT (LHRH agonist or LHRH antagonist according to physician’s choice) as first line treatment. Patient demographics and clinical features including age, ISUP grade group, prior treatment and distribution of metastases were obtained and documented. All patients underwent PSA-measurement and [^18^F]PSMA-1007 PET/CT for primary staging (PET1) before the initiation of apalutamide and at a second time point (PET2) during treatment with apalutamide. Baseline ADT was not initiated before PET1. Written informed consent was obtained from all participants. The study was approved by the Ethics Committee of the Medical Faculty of LMU Munich (# 19–0942).

### Radiopharmaceutical and imaging protocol

Patients received a mean activity of 214.6 ± 111.6 MBq (adjusted for body weight) of [^18^F] PSMA-1007 intravenously per PET scan, in accordance with previously reported radiosynthesis and administration procedures (1). Premedication with furosemide (20 mg) was administered to all patients without contraindications (2). The radiopharmaceutical was administered individually for each patient in compliance with the provisions of the German Medicinal Products Act, §13(2b). PET scans were performed from the skull base to the mid-thigh using a Biograph mCT scanner or a Biograph 64 PET/CT scanner (Siemens Healthineers Erlangen, Germany) 60 min after tracer injection (duration: 20 min; 2.5 min per bed position). PET/CT included a diagnostic, contrast-enhanced CT scan in the portal–venous phase (Imeron 350; 1.5 ml/kg body weight; Bracco Imaging, Milano, Italy). PET was reconstructed iteratively using TrueX (three iterations, 21 subsets) with Gaussian postreconstruction smoothing (2 mm full-width at half-maximum).

### [^18^F]PSMA-1007 PET analysis

Any focal uptake of [^18^F]PSMA-1007 that exceeded the surrounding background and was not associated with physiological uptake was considered to be suspicious for malignancy. The total tumor volume (TTV) was determined semi-automatically by applying a fixed threshold of 4.0 to the whole-body PET dataset (Hermes Affinity, Hermes Medical Solutions). The mean and maximum standardized uptake values (SUV_mean_ and SUV_max_) were evaluated before and after therapy initiation. TTV-SUV_max_ was defined as the maximum voxel SUV within the composite TTV mask. TTV-SUV_mean_ was defined as the average SUV of all voxels contained in the composite TTV mask. Additionally, post-treatment changes were analysed in accordance to the response evaluation criteria in PSMA PET/CT (RECIP) 1.0 [5]. No PSMA uptake on PET2 was defined as complete response (CR), while a decrease in TTV of ≥ 30% without new lesions as a partial response (PR). New PET-positive lesions on PET2 and an increase in TTV of ≥ 20% was classified as progressive disease (PD). Stable disease (SD) was defined as a decrease in TTV < 30% with or without new lesions or ≥ 30% with new lesions as well as an increase of < 20% with or without new lesions or an increase of ≥ 20% without new lesions.

### Statistical analysis

Statistical analysis was performed using SPSS 29.0 (IBM). Wilcoxon matched pairs signed-rank test was applied to determine significant differences between the groups. Descriptive statistics for groups are presented as median and ranges unless stated otherwise. P values below 0.05 were considered statistically significant.

## Results

### Patient characteristics

17 patients were included in the study, undergoing PSMA PET between May 2020 and January 2024. Patient demographics and treatment characteristics are summarized in Table 1. Patients with synchronous (12/17, 70.6%) and metachronous (5/17, 29.4%) metastatic disease were included. Most patients (64.7%) were classified as high volume disease according to CHAARTED criteria [15]. All included patients had bone metastases (M1b), 10/17 patients (58.8%) had non-regional lymph node metastases (M1a), no patient had visceral metastases.

**Table 1:** Patient demographics and treatment characteristics

Total number of patients	17
Median age (years, range)	72 (53–85)
Apalutamide medication Dose 240 mg/day, n (%)	17 (100)
ADT (LHRH agonist/antagonist), n (%)	17 (100)
Prior treatments, n (%)	9 (53)
Radical Prostatectomy	8 (47)
Radiation	1 (5.9)
ISUP Grade Group, n (%)	
2	4 (23.5)
3	4 (23.5)
4	3 (17.6)
5	6 (35.3)
Sites of metastases, n (%)	
N1	11 (64.7)
M1	
a	10 (58.8)
b	17 (100)
c	0 (0)
Tumor volume (CHAARTED [15]), n (%)	
Low volume	6 (35.3)
High volume	11 (64.7)
Risk category (LATITUDE [16]), n (%)	
Low risk	10 (58.8)
High risk	7 (41.2)
Oligometastatic disease (≤ 3 bone metastases), n (%)	6 (35.3)
Development of metastases, n (%)	
Synchronous	12 (70.6)
Metachronous	5 (29.4)

Participants received the androgen receptor pathway inhibitor (ARPI) apalutamide at a dose of 240 mg per day as first line treatment in mHSPC. No dose reductions or treatment discontinuations occurred during the analysed period. The average interval between PET1 and the initiation of apalutamide was 0.4 months (range 0.03–5.90 months) and between the initiation of apalutamide and PET2 5.30 months (range 1.70–20.30 months). The median interval between PET1 and PET2 was 5.60 months (range 3.30–20.60 months). All patients received baseline androgen deprivation therapy using LHRH agonists or antagonists according to physician’s choice, which were initiated in the range of a few days prior to or simultaneously with apalutamide.

### PSA response

Median PSA at PET1 prior to treatment with apalutamide was 46.40 ng/mL (range 9.20–289.00 ng/mL). All patients demonstrated a significant (*p* = 0.001) and positive PSA response following treatment initiation, with a median PSA level of 0.03 ng/mL (range 0.03–157.00 ng/mL) at PET2. Moreover, all patients achieved a PSA decline of more than 50% (PSA50) from baseline levels. PSA response is listed in Table 2.

**Table 2:** Changes in PSA, TTV-SUV_max_, TTV‑SUV_mean_ and total tumor volume between PET1 and PET2

	PET1	PET2	*P* value
Median PSA ng/mL (range)	46.40 (9.20–289.00)	0.03 (0.03–157.00)	*p* = 0.001
Median TTV-SUV_max_ (range)	28.3 (6.3–95.4)	11.6 (5.3-142.4)	*p* = 0.006
Median TTV-SUV_mean_ (range)	8.5 (4.3–23.7)	5.0 (4.2–32.2)	*p* = 0.020
TTV (range)	39.1 (5.4–1841.0)	14.9 (2.1–750.0)	*p* = 0.006

### PET metrics

#### TTV-SUV_max_

**Fig. 1:**
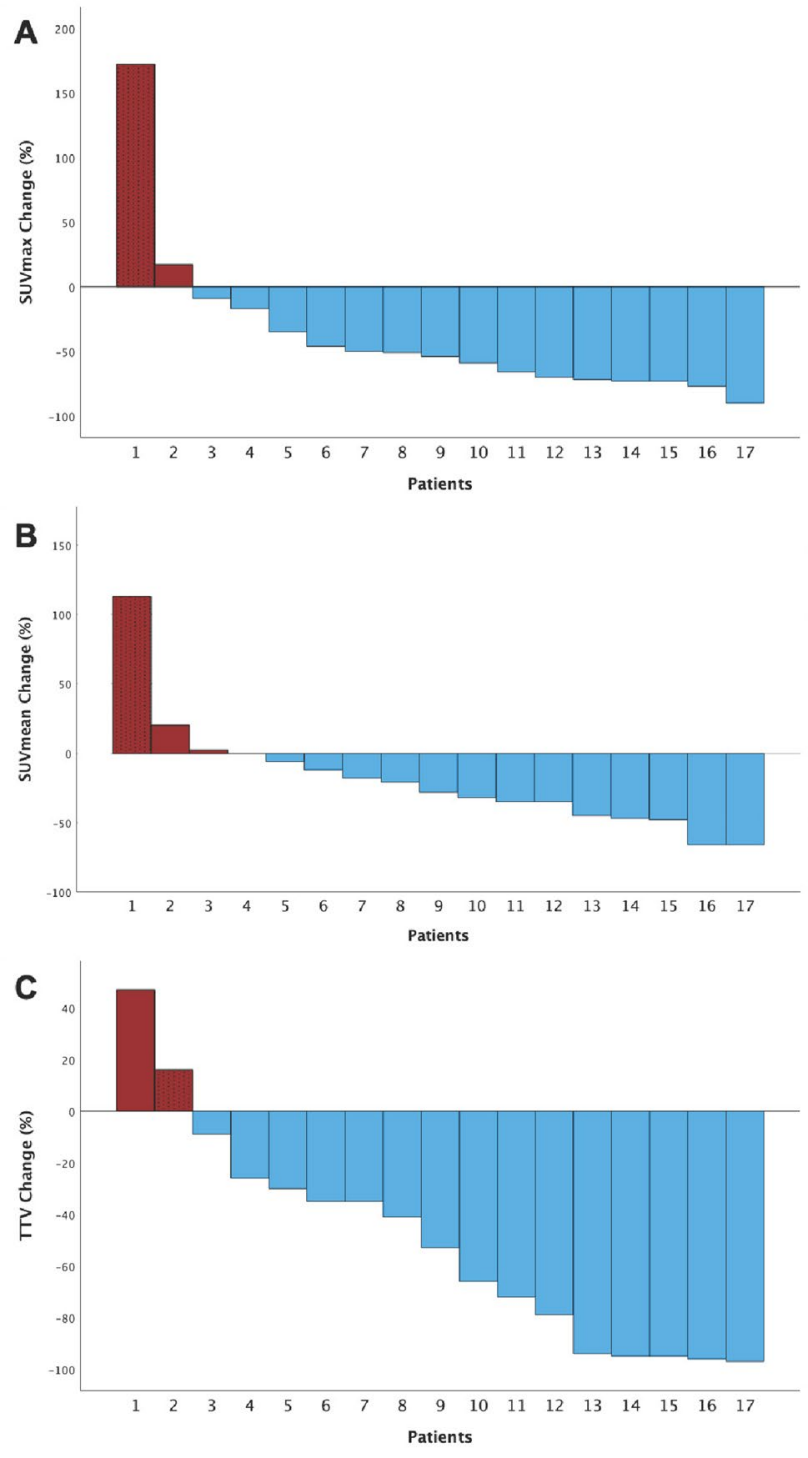
Waterfall plots displaying the extent of TTV-SUV_max_ (**A**), TTV-SUV_mean_ (**B**) and TTV (**C**) percent changes compared to baseline at follow-up PET2. Decreased parameters are marked blue, increased parameters are marked red. The dotted column equals the single patient exhibiting elevations of all three parameters

Median values and ranges of TTV-SUV_max_ before and after the initiation of apalutamide are shown in Table 2. A decrease in median TTV-SUV_max_ was observed in 15 out of 17 patients (88.2%; median change of − 59% (range − 90% - − 9%)) after the initiation of apalutamide, while 2 patients (11.8%) showed an increase (+ 17% (PSA: 9.20 ng/mL (PET1) vs. 0.03 ng/mL (PET2)) and +172% (PSA: 18.70 ng/mL (PET1) vs. 8.00 ng/mL PET2)). The overall median change in TTV-SUVmax was a decrease of − 54% (range − 90% - + 172%), which was statistically significant (TTV-SUVmax PET1 vs. PET2: 28.3 vs. 11.6, *p* = 0.006). Figure 1A illustrates the percentage change for each individual patient.

#### TTV-SUV_mean_

Median values and ranges of TTV-SUV_mean_ before and after the initiation of apalutamide are shown in Table 2. A decrease of TTV-SUV_mean_ was observed in 13 out of 17 patients (76.5%), three patients had an increased TTV-SUV_mean_ at PET2 (17.7%), while one patient (5.9%) showed no change in this parameter. The overall median change in TTV-SUV_mean_ was a decrease by -28% (range − 66% - + 113%). Among the patients with increased TTV-SUV_mean_ the median change was + 58% (range + 2% - + 113%). For patients with decreased TTV-SUV_mean_, the median change was − 35% (range − 66% - − 6%). Figure 1B depicts the percentage change observed for each patient.

#### Total tumor volume (TTV)

Median values and ranges of TTV before and during apalutamide are displayed in Table 2. A reduction in TTV was observed in 15 patients (88.2%), while two patients (11.8%) had an increased TTV (+ 16% and + 47%) at PET2. The overall median change in TTV was − 53% (range − 97% - + 47%). For patients with decreased TTV, the median change was − 66% (range − 97% - − 9%). Figure 1C shows the percentage change in TTV for each patient.

### RECIP 1.0

According to the RECIP 1.0 scoring system, 13/17 patients (76.5%) were classified as PR, 4/17 patients (23.5%) as SD and none (0%) as PD.

### Discordant response

**Fig. 2:**
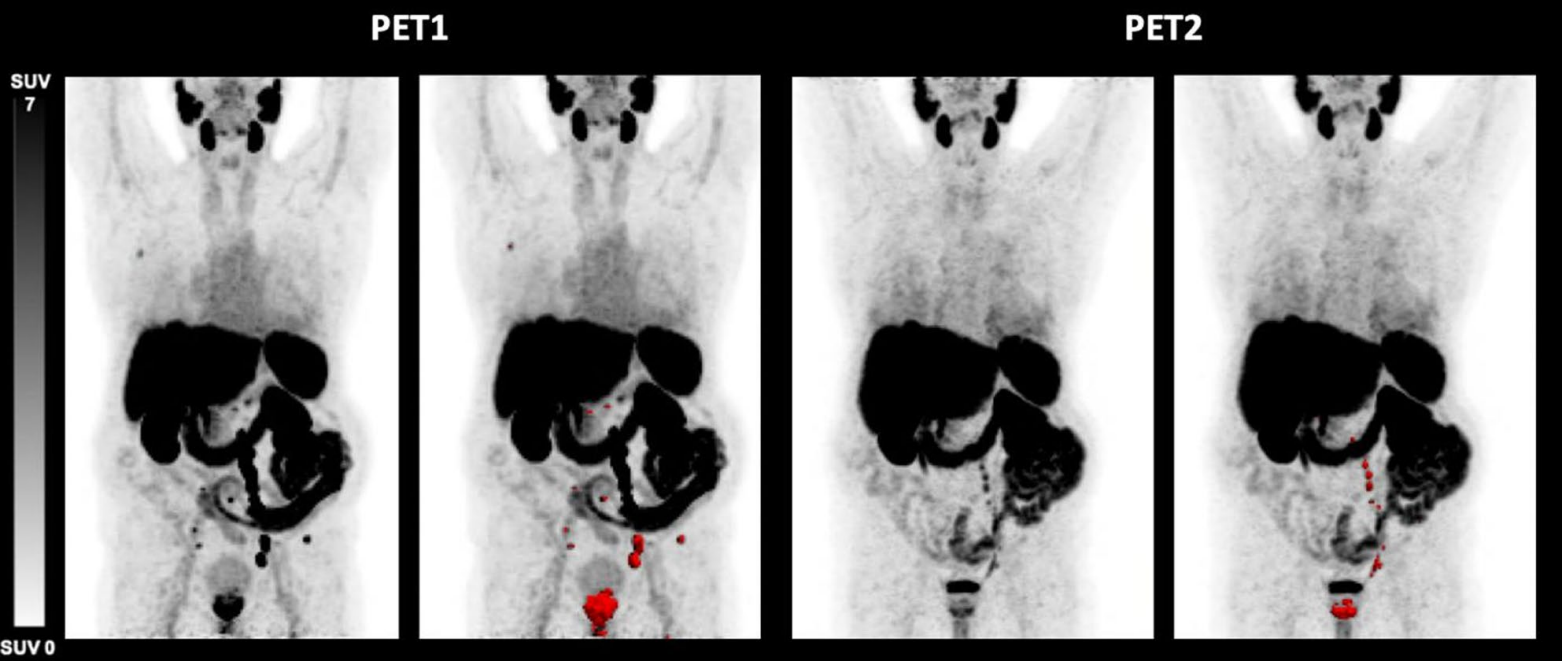
[^18^F]PSMA-1007 PET/CT derived maximum intensity projection (MIP) of a 79-year-old patient with metastatic prostate cancer before (PET1) and after (PET2) initiation of apalutamide therapy (time difference 5.00 months). TTV (− 34.7%) and SUV_max_ (− 58.6%) decreased, SUV_mean_ (+ 19.6%) increased, PSA level decreased from 25.00 ng/mL to 0.03 ng/mL. This patient was rated as PR according to RECIP 1.0

**Fig. 3:**
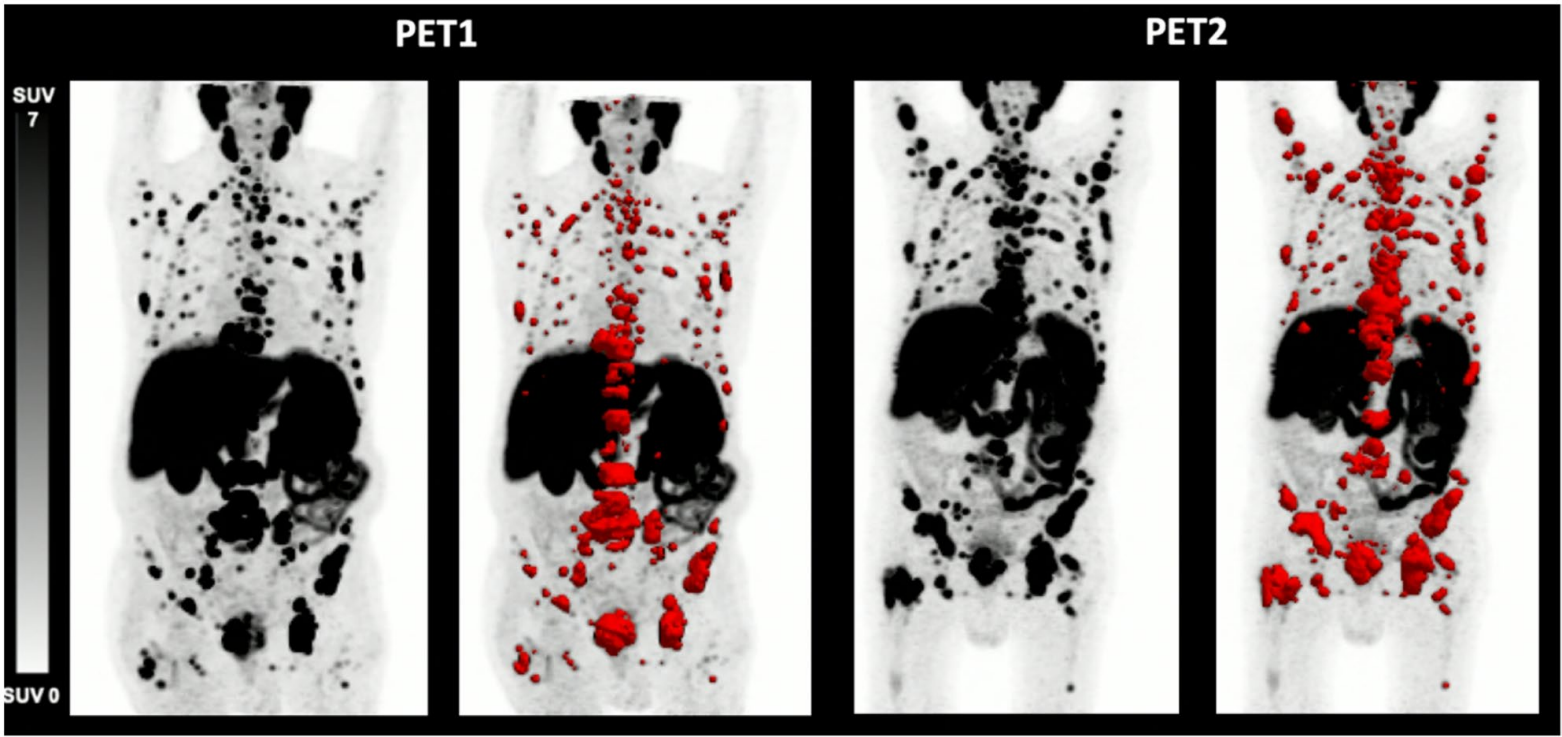
[^18^F]PSMA-1007 PET/CT derived maximum intensity projection (MIP) of a 75-year-old patient with metastatic prostate cancer before (PET1) and after (PET2) initiation of apalutamide therapy (time difference 1.60 months). TTV (+ 47.1%) increased, whereas SUV_max_ (− 34.6%) and SUV_mean_ (− 28.5%) decreased, PSA level decreased from 289.00 ng/mL to 55.00 ng/mL. This patient was rated as SD according to RECIP 1.0

Discordant response defined as elevations of either TTV, TTV-SUV_max_ or TTV-SUV_mean_ or a combination of these parameters despite biochemical response was seen in 4/17 (23.5%) patients. These patients showed at least one increasing imaging parameter at PET2 (TTV, TTV-SUV_max_, TTV-SUV_mean_), whereas the PSA level decreased (mean PSA at PET1: 85.50 ng/mL, mean PSA at PET2: 15.80 ng/mL). One patient (Fig. 2) had an increase exclusively in TTV-SUV_mean_ (9.7 vs. 11.6; 19.6%; PR), while another (Fig. 3) showed a rise solely in TTV (510 mL vs. 750 mL; 47%; SD). These two patients had synchronous disease, without prior treatments, and ISUP grade group 2 and 4.

**Fig. 4:**
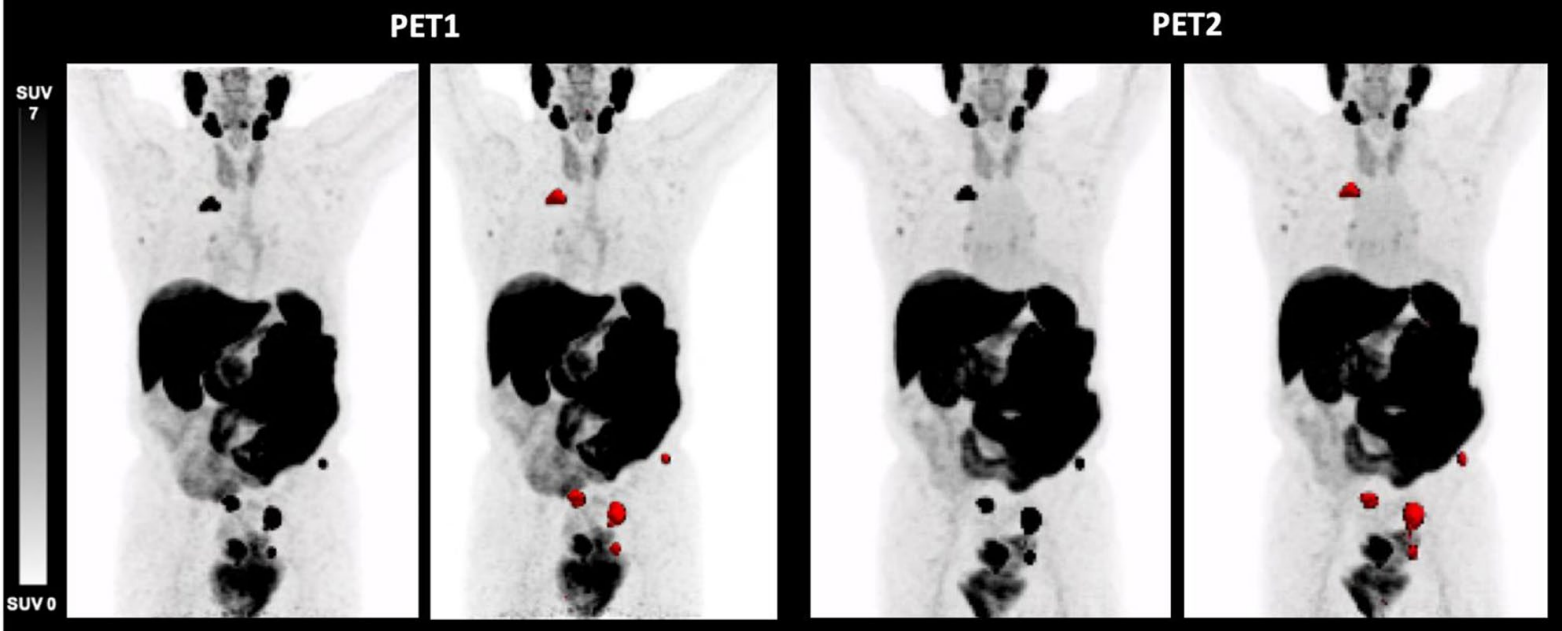
[^18^F]PSMA-1007 PET/CT derived maximum intensity projection (MIP) of a 85-year-old patient with metastatic prostate cancer before (PET1) and after (PET2) initiation of apalutamide therapy (time difference 2.70 months). TTV (+ 16%), SUV_max_ (+ 172%) and SUV_mean_ (+ 113%) increased, PSA level decreased from 18.70 ng/mL to 8.00 ng/mL. This patient was rated as SD according to RECIP 1.0

**Fig. 5:**
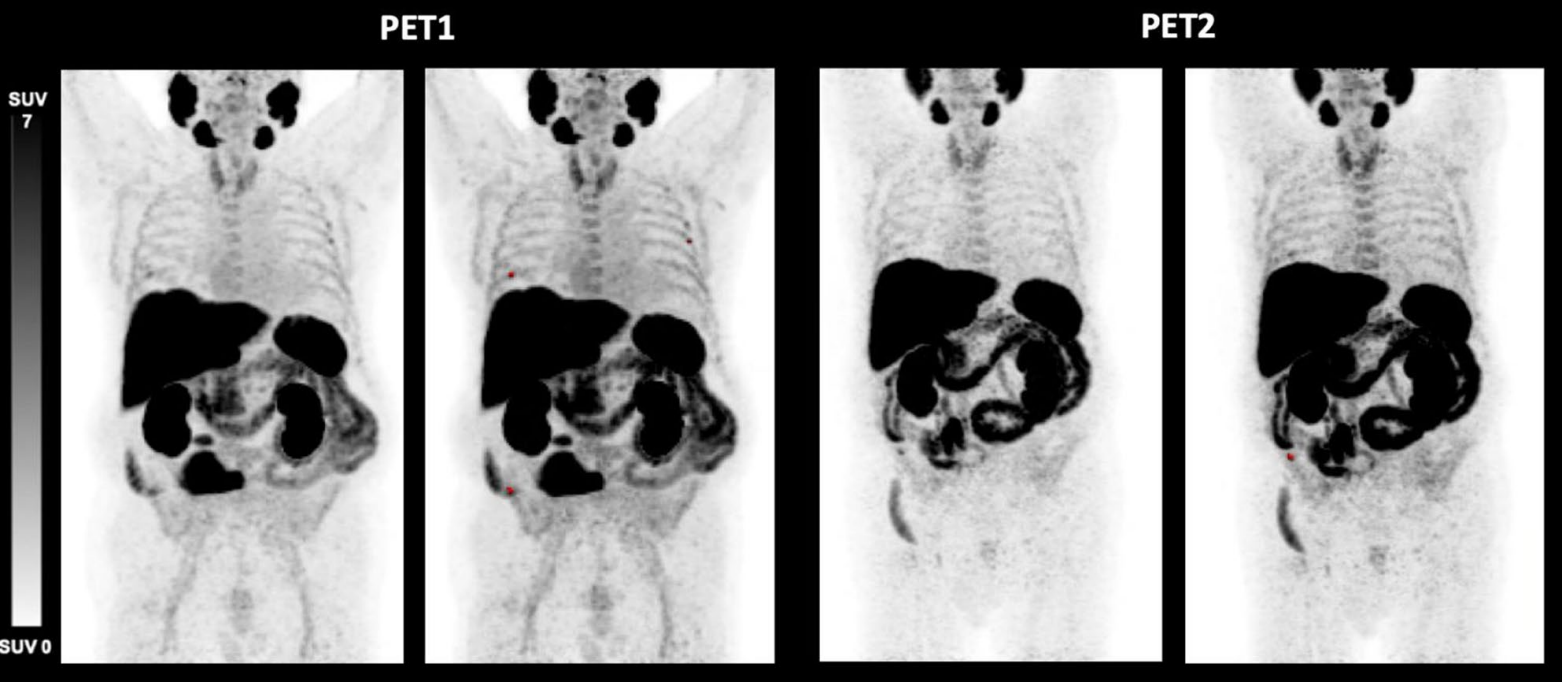
[^18^F]PSMA-1007 PET/CT derived maximum intensity projection (MIP) of a 78-year-old patient with metastatic prostate cancer before (PET1) and after (PET2) initiation of apalutamide therapy (time difference 8.00 months). TTV (− 9%) decreased, whereas SUV_max_ (+ 17%) and SUV_mean_ (+ 2%) increased, PSA level decreased from 9.20 ng/mL to 0.03 ng/mL. This patient was rated as SD according to RECIP 1.0

**Fig. 6:**
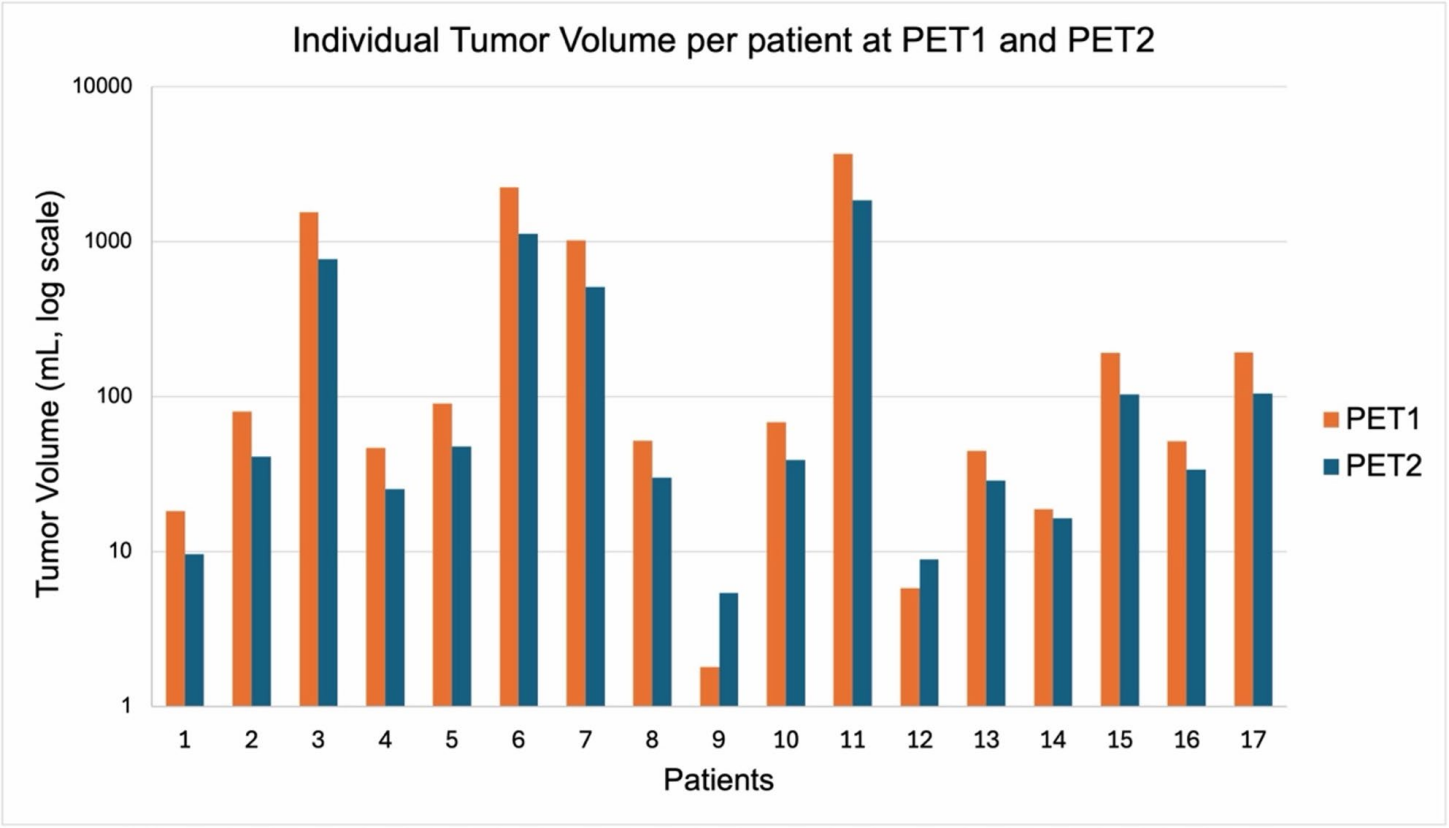
Logarithmic plot showing individual tumor volumes (mL) per patient (1–17) at baseline (PET1, orange) and follow-up (PET2, blue)

One patient (Fig. 4) demonstrated elevations of all three parameters (TTV: 28.8 mL vs. 33.3 mL (16%); TTV-SUV_max_ 52.4 vs. 142.4 (172%); TTV-SUV_mean_ 15.1 vs. 32.2 (113%); SD). Another patient (Fig. 5) presented with an increase of TTV-SUV_max_ (6.3 vs. 7.4; 17%) and TTV-SUV_mean_ (4.3. vs. 4.4; 2%) but stable TTV (9.6 vs. 8.7 mL; − 9%; SD). These two patients had metachronous disease and had undergone prior radical prostatectomy at an earlier stage, with histopathological ISUP grade group 5. Individual tumor volumes (mL) per patient at PET1 and PET2 are shown in Fig. 6.

The median time difference (PET1 to initiation of apalutamide) was 0.90 months in patients with an increase of at least one imaging parameter compared to 0.30 months for patients with a decrease (*p* = 0.35).

The median time difference (initiation of apalutamide to PET2) was 3.90 months in patients with an increase of at least one imaging parameter compared to 5.80 months for patients with a decrease (*p* = 0.20).

### Extended follow-up

Extended follow-up information after PET2, where available, is summarized in Table 3. Among the 4 patients who displayed an increase in at least one imaging parameter at PET2, two required changes in therapy to docetaxel or enzalutamide, one continued apalutamide with an adequate PSA response and one had unavailable follow up data. Of the 13 patients without increased imaging parameters at PET2, two showed clinical or biochemical progression in the extended follow up, resulting in a treatment switch to enzalutamide.

**Table 3:** Available extended follow-up information for the 17 patients included in the study, differentiating between patients with and without increased imaging parameters at PET2

Patient	Increased parameter at PET2	PET2 date	Follow up PET	Last FU date	PSA at last FU (ng/mL)	Therapy at last FU
1	Yes	Sept 2021	Mar 2022: SD	Mar 2022	0.03	Enzalutamide
2	No	Nov 2022	-	Oct 2025	0.03	Apalutamide
3	No	Dec 2021	Aug 2022: PD	Sep 2022	5.2	Enzalutamide
4	No	Sep 2021	Nov 2022: SD	May 2023	0.03	Apalutamide
5	No	Dec 2022	-	Jan 2023	0.03	Apalutamide
6	No	Jan 2022	May 2025: PD	Jul 2025	2.8	Enzalutamide
7	Yes	May 2022	-	Aug 2022	169	Unknown
8	No	Oct 2022	-	Oct 2022	0.57	Apalutamide
9	No	Dec 2022	Jun 2024: SD	Jun 2024	0.03	Apalutamide
10	No	Jun 2023	-	Oct 2025	0.21	Apalutamide
11	No	Mar 2023	-	Apr 2023	438	Apalutamide
12	No	Aug 2023	-	Oct 2025	0.03	Apalutamide
13	Yes	Jul 2023	-	Aug 2023	19	Docetaxel
14	No	Jan 2024	Apr 2024: SD	Oct 2025	0.03	Apalutamide
15	No	Sept 2023	-	Oct 2023	0.03	Apalutamide
16	Yes	Jul 2024	-	Nov 2024	0.03	Apalutamide
17	No	Jan 2024	-	Jun 2025	0.03	Apalutamide

The table displays the date of the last follow-up (FU), PSA level at last FU, FU PET results (where available) and ongoing therapy

## Discussion

In this single-center retrospective study, we aimed to determine whether specific changes in PSMA expression can be observed in patients receiving the ARPI apalutamide, a well-established and widely used substance for mHSPC. To our knowledge, this is the first study to explicitly focus on apalutamide-treated patients in this context. Our findings confirm that ARPI therapy has a significant impact on [^18^F]PSMA-1007 PET/CT imaging.

Several studies have reported an upregulation phenomenon, characterized by increased PSMA expression (SUV_max_, SUV_mean_) within days or weeks of ARPI/ADT initiation [13, 14]. Therefore, a PSMA upregulation would be expected in our cohort. Conversely, this was not observed in the majority of our included patients. Since the Phase III VISION trial reported that about 12% of patients with mCRPC were ineligible for PSMA-RPT due to low PSMA expression [6], this has prompted interest in mechanisms to enhance PSMA expression. However, our data could not show a PSMA upregulation as it has been detected in several previous trials. Only 4/17 patients had an increase of either TTV, TTV-SUV_max_ or TTV-SUV_mean_. This might be explained by the comparatively long time interval between apalutamide initiation and PET2. The median time difference (initiation of apalutamide – PET2) for patients displaying at least one increased PET parameter was 3.90 versus 5.80 months for patients without an elevation. Studies demonstrating a PSMA upregulation detected this phenomenon especially in an early follow-up imaging [9]. Therefore, our results likely reflect PSMA changes during intermediate to long-term treatment with apalutamide.

Additionally, preclinical versus clinical settings, castration status, ADT type and imaging protocols may lead to differing results when investigating a potential PSMA upregulation [13].

Our findings are consistent with previous studies that investigated PSMA expression during long-term ADT [17]. However, most previous studies utilized first-generation AR inhibitors or LHRH agonists rather than second-generation ARPI. Existing literature on PSMA expression under novel hormonal therapies remains controversial. Murthy et al. observed decreases in tumor volume and SUV_mean_ over time with various ADT/ARPI combinations [11]. Conversely, Sonni et al. reported heterogeneous results at 1 week and 3 months after ARPI (enzalutamide, abiraterone or apalutamide) initiation in mCRPC patients, suggesting variability in response to androgen receptor modulation [10]. Rosar et al. found PSMA upregulation in mCRPC patients during short-term enzalutamide use and proposed it as a potential enhancer for PSMA-RPT [12].

All patients in our cohort exhibited a PSA decrease, consistent with favourable treatment response in first-line mHSPC patients. Apalutamide is known to induce a rapid and deep PSA response, which was confirmed in this study, with a median PSA level at PET2 of 0.03 ng/mL and a PSA decline of > 50% in all patients. However, four patients showed a discordant response with a significant decrease in PSA despite an increase in at least one imaging parameter. It is therefore possible that PSMA PET may detect progression earlier than PSA.

The heterogeneity in response could be influenced by the clinical setting, such as baseline PSA levels, prior local treatments, ISUP grade group, and the timing of PET acquisition. Notably, patients with isolated increases in TTV-SUV_max_ or TTV-SUV_mean_ had a history of radical prostatectomy and high ISUP grade group. A consensus on PSMA expression changes under ARPI/ADT based on castration status has yet to be established. Aggarwal et al. found increased PSMA uptake in most of both mHSPC and mCRPC patients, while Emmett et al. reported a reduction in [^68^Ga]-PSMA-11 intensity in 86% of mCSPC patients within days of ADT initiation, contrasting with an increase in mCRPC patients during the same period [14, 18]. Emmett et al. attributed the decrease in mHSPC primarily to high antitumor activity of ADT in hormone-naïve patients.

Our results align with these findings, as most patients demonstrated a significant reduction in PSMA expression. It is important to note that mHSPC and mCRPC represent distinct biological and clinical disease stages, which may contribute to the variability seen in previous studies.

This study has limitations, including the small sample size and heterogeneity in PET2 timing, which is also caused by its retrospective design. The relatively long interval between PET1 and PET2 may have obscured the proposed upregulation phenomenon after ARPI initiation, which typically occurs within days to weeks. Another limitation is the absence of image heterogeneity analysis. The applied PET parameters may not fully represent intratumoral variation, which could contribute to differences between imaging and clinical response. While parameters such as TTV, SUV_max_, and SUV_mean_ represent current quantitative standards in PSMA PET reporting, future investigations in larger patient cohorts should incorporate radiomics-based heterogeneity analyses to better capture the biological complexity of disease and to explain discordant imaging and clinical response patterns.

The majority of patients in this cohort demonstrated an imaging response, indicating that a PSMA upregulation is not commonly observed after intermediate to long-term apalutamide treatment. However, a small subset of patients exhibited an increase in PSMA PET parameters despite a decrease in PSA levels. This finding suggests that while PSMA upregulation in this setting appears uncommon, it cannot be completely excluded in individual cases and might reflect early imaging progression rather than a biological upregulation phenomenon. Nevertheless, long-term data are lacking.

## Conclusions

This pilot study demonstrates a significant decrease of PSMA expression as well as PSA levels among most mHSPC patients receiving apalutamide. These findings indicate that PSMA upregulation is unlikely during intermediate to long-term apalutamide therapy in the mHSPC setting. Nevertheless, a subset of patients displays an increase in imaging parameters despite PSA decline, which should prompt careful interpretation and close follow-up to rule out early disease progression.

## Data Availability

The datasets used and/or analysed during the current study are available from the corresponding author on reasonable request.

## Abbreviations

ADT: Androgen deprivation therapy
ARPI: Androgen receptor pathway inhibitor
CT: Computed tomography
mCSPC: metastatic castration-sensitive prostate carcinoma
PET: Positron-emission-tomography
PSMA: Prostate specific membrane antigen
RPT: Radiopharmaceutical therapy
TTV: Total tumor volume
TTV-SUV_max_: Maximum standardized uptake value of the total tumor volume
TTV-SUV_mean_: Mean standardized uptake value of the total tumor volume
WB: Whole body
